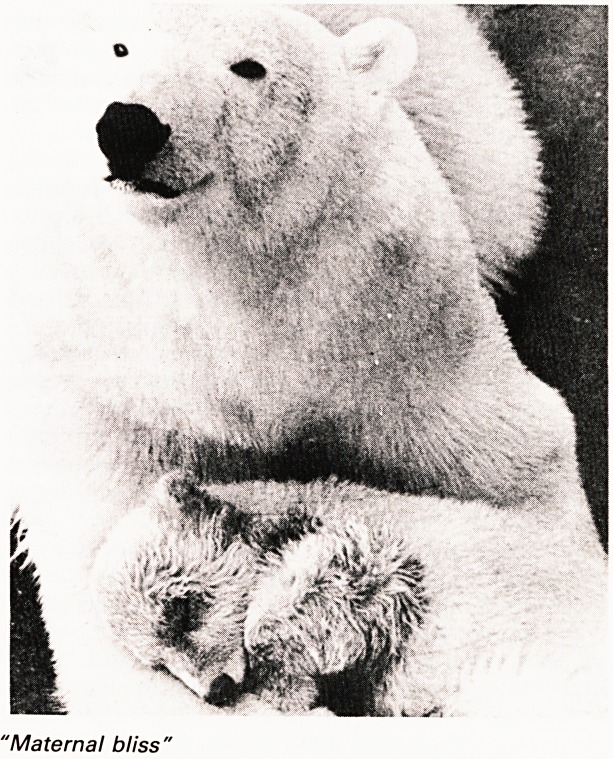# Presidential Address, Medicine and the Bristol Zoo

**Published:** 1982

**Authors:** Robert P. Warin

**Affiliations:** Consultant Dermatologist, United Bristol Hospitals


### Medicine and Bristol Zoo

**Robert P. Warin**
Consultant Dermatologist, United Bristol Hospitals

*Presidential address October 1981*

Dr. A. J. Harrison was President of the Medico-Chirurgical Society in 1894, some 20 years after its origins, and later it will be clear why I mention him.

He started off his Presidential address with the words, "Had I not your goodwill and wishes to encourage me, I might well feel appalled to occupy the Presidential chair this evening, when I reflect upon the stirring and clever addresses which adorn the archives of Bristol Med-Chi Society".

Contemplating a further 87 years of Presidential addresses, including that of Norman Brown last year, I feel even more appalled and not a little apprehensive. Nevertheless I would like to thank you for this great honour.

As you will know, the custom is for the inaugural address to be about some general subject and my talk tonight is on "Medicine and Bristol Zoo". The purpose is to look at the history of our Zoological Gardens, particularly in relation to the very considerable part played by Bristol doctors.

I have been on the Zoo Council for many years and would welcome tonight our present President, Mr. Richard Strachan, some members of the Executive Committee and the Director, Mr. Geoffrey Greed.

### EARLY YEARS

London Zoo was founded in 1831, partly from the Royal menagerie founded by Henry I at Woodstock, and the few remaining animals in the Tower of London. The Bristol, Clifton and West of England Zoological Society was founded in 1835. John Miller, the owner of the nursery garden off Whiteladies Road and now Garraways, formed the plan for the Zoo to be built on land at Arno's Vale, but this suggestion was then discarded in favour of the estate of Summer Trenmoor near Clifton Down turnpike gate and Gallows Acre Lane.

In 1808 the Bristol Institution was founded from the Philosophical and Literary Society. This was located at the bottom of Park Street, which is now the Masonic Hall. There were lectures, publications and exhibits of a remarkable standard. For example, the first gorilla's skull brought to Europe was disected and described and compared with a skull of a chimpanzee. This took the form of a 50-page monograph with remarkable lithographic prints.

In 1823 the formation of the Museum and the Zoological Gardens was proposed by influential and energetic members of the Bristol Institution, but it was not until 1835 that the Zoological Gardens were in being.

### A LOOK AT CONTEMPORARY EVENTS

| | |
|---|---|
| 1811 | Assembly Rooms opened in the Mall. |
| 1830 | Launch of the Great Western. |
| 1831 | Riots of Bristol. |
| 1831 | General Hospital opened. Rebuilt 1852. |
| 1928–1938 | Racecourse on Durdham Down |
| 1833 | Medical School (affiliated to University College 1876). |
| 1834 | Slave owners compensated. (6 of the original Zoo proprietors were compensated for freeing their slaves). |
| 1841 | Great Western Railway – Bristol to London. |
| 1842 | Royal Agricultural Show held behind Victoria Rooms. |
| 1842 | Suspension Bridge (Clifton abutment). Completed 1864. |
| 1843 | *Great Britain* launched. |
| 1849 | Cholera epidemic. 444 deaths. |
| 1862 | Clifton College. |

At a public meeting a proposition was moved by Dr. Henry Riley "that this meeting being persuaded that a Zoological Society in Bristol and Clifton will tend to promote the diffusion of useful knowledge by facilitating observation of the habits, form and structure of the animal kingdom as well as affording rational amusement and recreation to the visitors of the neighbourhood". The original proprietors were 220, including famous Bristol names of Fry, Pountney, Pinney (the Lord Mayor who escaped over the roof of the Mansion House in Queen's Square at the time of the Bristol riots), Sturge, Wills, Goldney. Bush, Harford, Brackenridge, Worral, Tyndall, Duke of Beaufort (present Duke's grandfather), also I. K. Brunel and many Bristol doctors including John Beddoe.

*Dr. Henry Riley*

### DR. HENRY RILEY

One of the main driving forces behind the beginning of the Zoo and its first secretary was Dr. Henry Riley. Born in Windsor Terrace in 1797 and trained in London and Paris he became a physician to the B.R.I. in 1834. Riley lectured at the School of Anatomy and the Bristol Institution on anatomy, zoology, philosophy and fossils. He had discovered fossils of rhinoceros, hippopotamus, etc., in some workings opposite Upper Belgrave Road and it is amusing to think of these animals wandering about outside what has now become the Zoo. In the B.R.I. records there is a complaint by a "dresser" that Riley ordered too many bleedings and that the row of patients requiring this treatment was too long to be carried out in one session. It was claimed that he was the first to use a stethoscope in the West Country and he would be introduced to this by Laennec in Paris.

He lived in Berkeley Square and became engaged to Cecilia, the daughter of a B.R.I. surgeon who also lived in Berkeley Square. He failed to turn up for the ceremony and there is a graphic account in the *Bath Herald* (January 5th, 1833) headed "Matrimonial disappointment". "At the appointed hour of eight everyone was assembled in holiday dress – with one exception – the intended bride in the freshness of her virgin beauty stood on the tiptoe of expectation – alas! for her no spouse prepared the bridal ring. The gentleman was sent for, but in spite of the father's remonstrances and the lady's tears, refused to ratify his engagement, under the plea that it was too early an hour for such an important event. No small quarrel in consequence arose between the parties, and eventually terminated in a final separation. Who shall decide when doctors disagree?"

He must have been a truly remarkable man and indeed she a remarkable lady, as two years after this they were married at St. George's, Brandon Hill.

He was one of the doctors connected with "body snatching" for disection and indeed he was caught in Brislington cemetary and fined £6 for this activity.

Along with a group of other Bristol doctors Henry Riley attended the first B.M.A. meeting in 1832 at Worcester, and he was on the first B.M.A. Council. In 1833 the first Annual Meeting was held in Bristol and Riley then "entertained" the delegates with a paper on "A description of the Anatomical structure of the liver of the rat from Cuba". He died (aged 51) in 1848 from a brain tumour.

He must have been a man of great personality and enthusiasm and was a driving force behind the beginning of the Zoo and then held the office of its first secretary.

### ORIGINS

The finance of the Zoo at its origin is of interest. The land cost £3,500 and the buildings and gardens a further £3,500. The weekly wage bill was £5.16.6d. The layout of the gardens have altered little over the years as is shown in the plan of 1866.

*Plan of the Gardens 1886*

The Senior Honorary administrator was initially the Treasurer and more recently, the President. The following is a list of the Treasurers/Presidents since 1836.

| | |
|---|---|
| 1836–1850 | William Goldney. |
| 1850–1858 | S. H. Stedder. |
| 1858–1873 | A. H. Knapp. |
| 1873–1882 | **Dr. W. C. Trotman.** |
| 1882–1884 | S. Lang. |
| 1884–1886 | Robert Coles. |
| 1886–1908 | **Dr. A. J. Harrison.** |
| 1908–1914 | His Honour J. V. Austin. |
| 1914–1921 | **Dr. A. J. Harrison.** |
| 1921–1924 | A. McArthur. |
| 1924–1928 | **Colonel J. P. Bush.** |
| 1928–1929 | **Dr. R. C. Clarke.** |
| 1929–1950 | W. S. Clarke. |
| 1950–1966 | J. S. Young. |
| 1967–1972 | **H. L. Shepherd.** |
| 1972– | R. G. Strachan. |

Little is known of Dr. Trotman who lived at 5 Saville Place and we now come to Dr. A. J. Harrison who took office in 1886 and then with a short break just before the first world war, carried on till 1921 when he was aged 85.

### Dr. A. J. HARRISON

Dr. A. J. Harrison was born in 1836 in Staffordshire and came to Clifton in 1877, having been trained at Birmingham and Guy's Hospitals. In 1879 he was elected Assistant Physician (including skin diseases) at the General Hospital and in 1901 he became the first dermatologist. He was President of the Bristol Medico-Chirurgical Society in 1894 and his inaugural address was on "Comparative Pathology". In 1901 he was President of the Dermatological Society of Great Britain and Ireland and in 1906 was the Long Fox Memorial lecturer. Apart from his administrative committments to the Zoo over this long period, he also had a great interest in the welfare of the animals and carried out many of the treatments and all the post-mortems himself. Many interesting stories were reported about this time.

The cape ostrich died with a grossly distended stomach and gizzard. At autopsy the contents contained eight to nine pounds of cabbage leaves, young grass, Indian corn, glass buttons, buckles, safety pins, pieces of glass and metal, a pen holder, and two pocket handkerchiefs with a name on one, a memorandum book and, as a last straw, the remains of a prayer book.

*Dr. A. J. Harrison*

The subject of foreign bodies in stomachs of animals is a very interesting one. One of the seals had ten pints of stones weighing 22 lbs. This is quite a well-known finding by seal hunters and it has been wondered if they acted as a sort of ballast or as an aid to food digestion.

The lion, Hannibal 1st, aged 17 was found dead. Autopsy showed a grossly enlarged spleen which had ruptured with much extravasation of blood. Apparently he had mated with the lioness on the previous day at 10.00 and again at 18.00 hours, and presumably this had led to the splenic rupture. One feels very sorry about the male lion dying in this way, but the other side of the coin concerns a female llama, 10 years old, who suffered from a "love bite" in the neck by the male and the jugular vein was opened and she died.

Sixteen weeks after Hannibal died the lioness produced 3 fine cubs. One of these cubs was Jupiter, who had a long period of connubial bliss which produced 26 cubs. He showed hydatid cysts of the liver on autopsy. He also at one time had an in-growing claw removed by Blunsden, the famous Head Keeper, with pincers after being put in a crush cage. Dr. Harrison himself extracted a tooth from a year old tiger in a similar "sophisticated" way. The tiger was in a sack and a roll of wood was placed in it's mouth and held from behind by the long-suffering keeper.

The history of anaesthesia in Zoo animals is a fascinating study and we now have in Bristol a recognised expert in this field, Dr. Barbara Weaver. Dr. Harrison tried giving chloroform in 1890. One of the eye surgeons came along to help with the removal of a cataract from the cockatoo, but the response of the bird to the chloroform was some quick flaps, followed by expiry, and Dr. Harrison was not keen on anaesthetics after this experience. However in 1895 chloroform was successfully given to an Arabian Gazelle when Mr. Munro-Smith performed a Caesarian section.

The Russian bear, Judy, had two cubs and after this had no food or drink for 75 days, supporting the cubs on her own fat. The subject of fasting has recently been of great importance but in 1894 Dr. Harrison made the comment that "None of our fasting heroes and heroines can hold a candle to Judy". In a similar vein pythons will often have long fasts. One large reticulated python had a duck at Christmas 1880. His next meal was on June 21st but following this he fed regularly about once a week. One python lived for eighteen months without food. Concerning snakes, Dr. Harrison successfully opened an abcess on a black constrictor snake with his pen-knife.

There are a lot of other stories connected with Dr. A. J. Harrison. He knew all the animals so well and had trained the chimpanzee to unbutton his gloves when he came to the Zoo.

*Lifeboat after launch in Zoo lake 1872*

A curious phenomenon was the launching of lifeboats on the lake. The first boat launched in 1867 was named *The Bristol and Clifton.* It was sent by train to Lossiemouth and saved a large number of lives. The second was launched in 1872, named *Jack a Jack*, and was largely subscribed by Bristol Merchants who were losing so many ships and men on the rocky North Devon coast. It then went to Morte Point, being towed by steamer to Ilfracombe. On both occasions some 60,000 people lined the streets up to the Zoo and there were bands, cavalry, naval personnel and general celebrations.

### GENERAL AFFAIRS

At the present time the Zoo is financed from the gate money and regular subscriptions. It receives no help from national or City finance and indeed is a very heavy rate payer. In the late nineteenth century century and early twentieth century fetes were an important source of income and these included concerts, dancing, tennis, croquet, archery, skating and firework displays. For example, the list of fetes in 1899 included the Zoo band, the Cathedral school annual sports, a Carnival in aid of the B.R.I. Nurses' Home extension, and a firework display celebrating the Battle of Omdurman, etc., etc. During the first Great War a lot of entertainment for the troops and wounded was carried out in the Zoo, but many of the animals were disposed of. One of the seals joined the Navy. He went to Portsmouth and the idea was to feed him from a submarine and he would then learn to sniff out U-boats and so seal their fate! A failure and the seal deserted.

*Col. J. Paul Bush*

### Col. J. PAUL BUSH

Returning now to the medical Presidents we come to Colonel J. Paul Bush, 1924–1928. He was a surgeon at the B.R.I. from 1885 to 1913 and died in 1930. During the South African war he had been surgeon in charge of a field hospital (awarded C.M.G.) and in 1914 became Commandant of the second Southern General Military Hospital, which was based on the King Edward VII Memorial building at the B.R.I. and also Southmead Hospital (awarded C.B.E.). His grandson, Robin Bush, is at present on the Zoo Council.

### Dr. RICHARD CLARKE

Many of us remember Dr. Richard Clarke and I once had the privilege of going as his guest to one of the Bristol Medical Dinners and was completely enthralled by his exuberance and infectious enthusiasm together with his fascinating talk about the Bristol medical scene and the Zoo. Richard Clarke was President 1928–1929. He had, however, been a member of the Council for over 30 years and indeed was the main driving force behind the Zoo for many years. He was born in 1876 and died in 1957 (aged 71). Trained at Clifton College and Bristol University he became honorary physician to the Royal Infirmary in 1913. He had a remarkable military service, being in the territorial army (Bristol Rifles) in 1904. During the first Great War he served in Casualty clearing stations from whence came an O.B.E. and his wife who was a Q.A. sister. In the second Great War he served as a Commandant to a General Hospital in the Middle East until 1941.

Just after the first Great War the Zoo went through very difficult times financially and indeed it is reported that Clifton College was measuring up the grounds to see how many football pitches could be accommodated. Richard Clarke, together with his cousin Sefton Clarke, and on the business side, Mr. Burgess, transformed the Zoo with Mr. Greed, whom they appointed as superintendent in 1928, and who was later made Director. Their combined skills and enthusiasm brought the Zoo back to being a scientific society, showing all sorts of interesting animals and financially successful.

Richard Clarke designed the Aquarium, the Monkey Temple and the old nocturnal house. He had all sorts of progressive ideas about keeping the

*Dr. Richard Clarke*

*Alfred (famous Bristol citizen)*

animals healthy and was closely associated with Alfred, the first gorilla who became one of Bristol's best known citizens. He was worried about droplet infection and indeed Alfred at one time had whooping cough and it is for this reason that these animals are now largely behind glass. He diagnosed a thyroid deficiency in Alfred and treated him with thyroid. He also gave him Vitamin D. because the black skin might limit Vitamin D. synthesis. This was all reported in the Med-Chi Journal. Alfred finally died of tuberculosis in 1948. Tuberculosis was at one time a real problem and a number of chimpanzees, orang-utans and monkeys have died of tuberculosis.

Apart from Richard Clarke the whole Clarke family has given outstanding service to both the Bristol Hospitals and the Zoo. His brother, Cyril Clarke, served on Management Committees at the B.R.I. from 1911 until 1953 and was Chairman of the Board of Governors from 1944 to 1953. Dr. Clarke's nephew is Charles Clarke who was Chairman of U.B.H. 1968 to 1974 and has continued to play such an important part in contemporary Bristol medical organization. Dr. Clarke's son-in-law was Malcolm Campbell who was our well-loved consultant neurologist at the B.R.I., and his daughter is Suzanne Clarke, our consultant virologist.

### RECENT DEVELOPMENTS

It is interesting to consider the various developments which have occurred in the Zoo in recent times and the following is a list of the main ones since 1950.

| | |
|---|---|
| 1950 | Reptile House. |
| 1951 | Penguin Enclosures. |
| 1953 | First Nocturnal House. |
| 1961 | Birds of Prey Aviary. |
| 1966 | Hollywood Tower Estate. |
| 1969 | Free Flights Bird House. |
| 1971 | New Zebra House. |
| 1972 | New Services Building. |
| 1976 | New Ape House. |
| 1977 | New Cat Complex. |
| 1981 | New Reptile House. |
| 1981 | New Nocturnal House. |
| 1981 | Children's Corner. |

### Mr. H. L. SHEPHERD

We are coming now into more easily remembered times and our much admired Harry Shepherd was President of the Zoo from 1967 till 1972. He had,

*Mr. H. L. Shepherd*

however, been on the Zoo Council and Honorary Obstretician to the Zoo since 1935. He came to the B.R.I. in 1924 and became Consultant Obstretician and Gynaecologist in 1931. Besides being an excellent surgeon he was a superb administrator and held administrative posts in the United Bristol Hospitals, South West Regional Hospital Board and at Frenchay Hospital. He was awarded an honorary M.D. (Bristol) in 1972, and was Secretary and President of the Medico-Chirurgical Society. Harry Shepherd played rugby for Bristol, was a scratch golf player and had a wonderful enthusiasm for all these varied activities. He played a big part in the Zoo organisation but also helped to produce a remarkable record of births in the Zoo. His gynaecological was nibbled by a tiger foetus.

Adam was the first chimpanzee born in captivity. There was close link of baby primates and the hospitals and in 1957 one was for a short time in an incubator in the Maternity Hospital. Not the first time the hospital has helped out in such a way, as Richard Clarke managed to admit two baby bears with pneumonia to the Children's Hospital where they were treated in a steam tent.

When thinking in terms of baby primates, I must mention Dr. Beryl Corner who has devoted hours of her time and valuable expertise to rearing these babies.

*Adam – first chimpanzee born in capitivity*

*"A zoo family" or "A long way to grow up"*

I like to think I have helped a little but the following is not to my credit. I was asked to deal with a wart on the upper lip of one of the orang-utans. After much consideration I presented myself to the orang-utan with an applicator dipped in pure trichloracetic acid and having made an expert approach but just before the point of contact, quick as lightning, he grabbed the stick and swallowed it. It was rather a worried dermatologist who visited the animal for the next few days but he seemed perfectly happy and one week later the wart dropped off. I have never discovered whether Harry Shepherd really knew the whole story, but at the next animal committee meeting he expressed his delight that I had dealt with the wart in such an expert way!

At one time the animal committee used to spend much effort thinking up names for the main animals and it is a curious coincidence that when the baby female rhinoceros was named, our very excellent, efficient and well-loved B.R.I. matron was also named Rhona. The male rhino developed widespread eczema and we treated him with large tubs of slightly out-of-date Betnovate. It was entirely due to psychological stress of a sexual nature, but it would be a pity to bore you with the details.

### RESEARCH

Valuable research has been carried in the Zoo, particularly in conjunction with the University and we hope that a lot more will be done. Just to mention a minor piece of not-too-serious research concerning the rhesus monkeys who were in a dreadful state due to hair pulling, bites and other injuries from bullying and general nastiness. The Department of Psychology set up an in-depth study

*"Do be careful with that baby"*

and after some six weeks, they wrote a really fascinating report on the psychological aspects. It was not very clear how the problem should be dealt with but the final treatment was entirely effective – we got rid of the lot and started with a colony of a new species. How often you must have wished to indulge in similar treatment for your patients.

In conjunction with the post-mortem collection, I must mention Professor Tom Hewer's work in the Zoo. He has been the Honorary Pathologist since 1939 and there is now an immense amount of careful pathology work stored up in the files. He has given a large amount of his time and energy to the Zoo and indeed when only eighteen months old, contributed the tip of one finger attached to a peanut.

You will remember that Dr. Henry Riley was fined for "body snatching" round about the time of the inception of the Zoo. When Professor Hewer arranged to do the autopsy on the gorilla, Alfred, he was horrified to find that the body had been spirited away and was being shared out amongst anatomists in the Medical School. The acquisitive enthusiasm turned to panic when it became known that Alfred had died from tuberculosis. Professor Hewer's advice on the gardens, animals and new buildings will long be remembered.

Dr. Bradbeer has helped a lot with anaesthesia in the past. Doctors Peter Dunn and George Foss are a great help and their expertise with neo-nates and reproductive problems is very valuable. Mr. Robert Horton's quiet commonsense is now in demand on the Executive Committee.

However veterinary science has reached such a state of expertise that the medical amateur must now hand over to our really excellent veterinary surgeons, but I like to think that we have a part to play in the Zoo in many ways.

*"Maternal suspicion"*

*Somebody's darling*

*"Maternal bliss"*

## Figures and Tables

**Figure f1:**
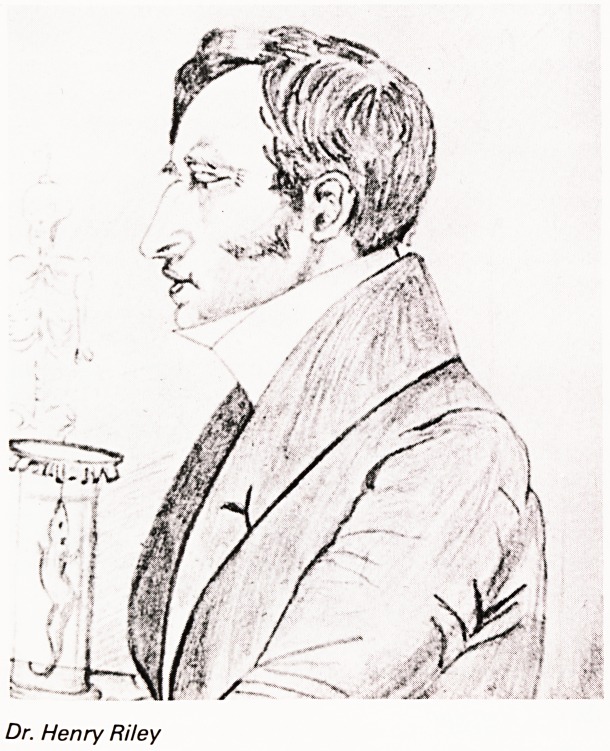


**Figure f2:**
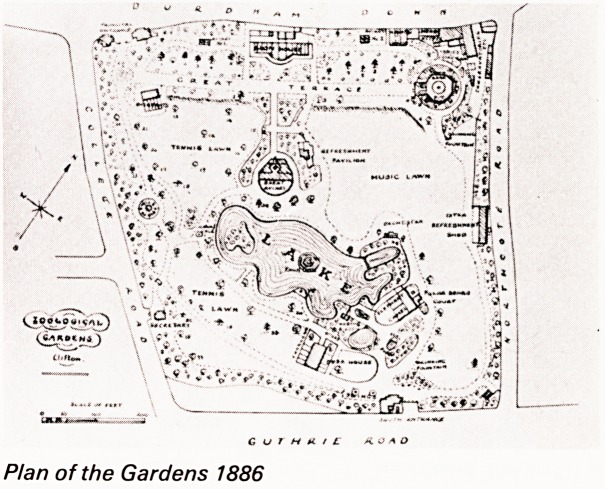


**Figure f3:**
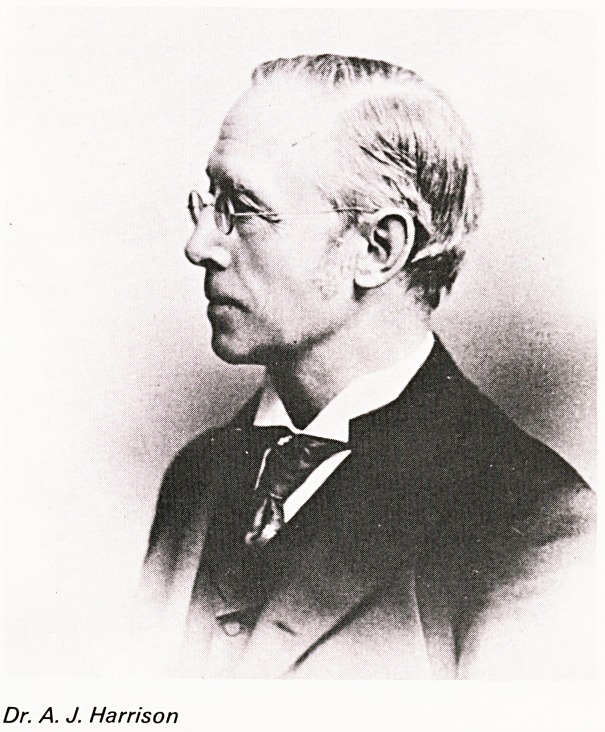


**Figure f4:**
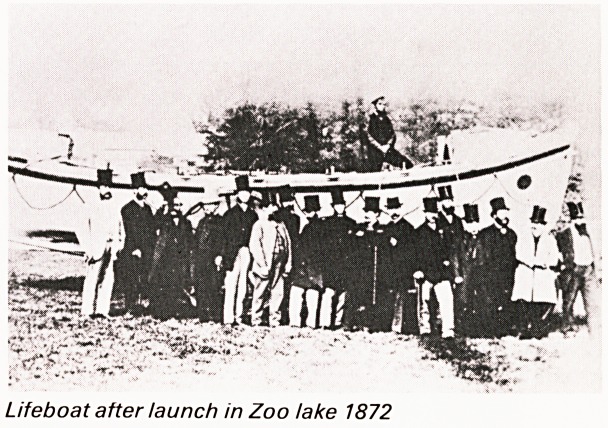


**Figure f5:**
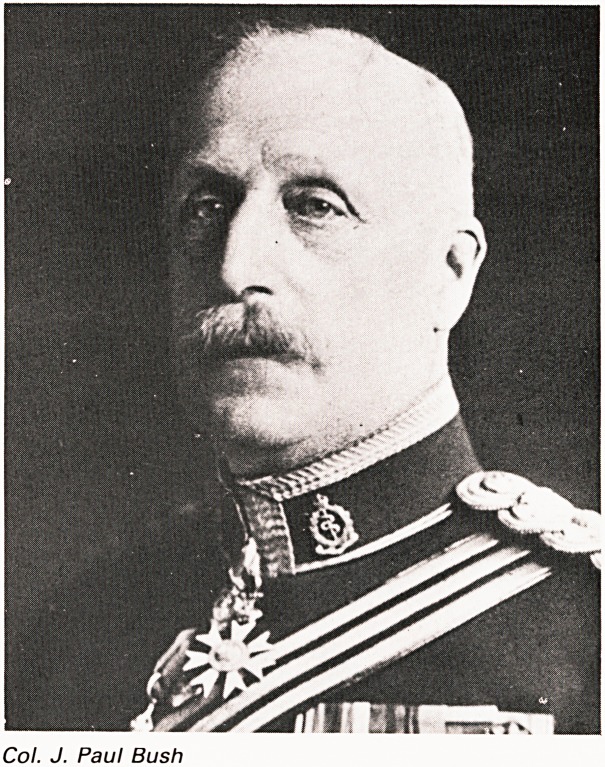


**Figure f6:**
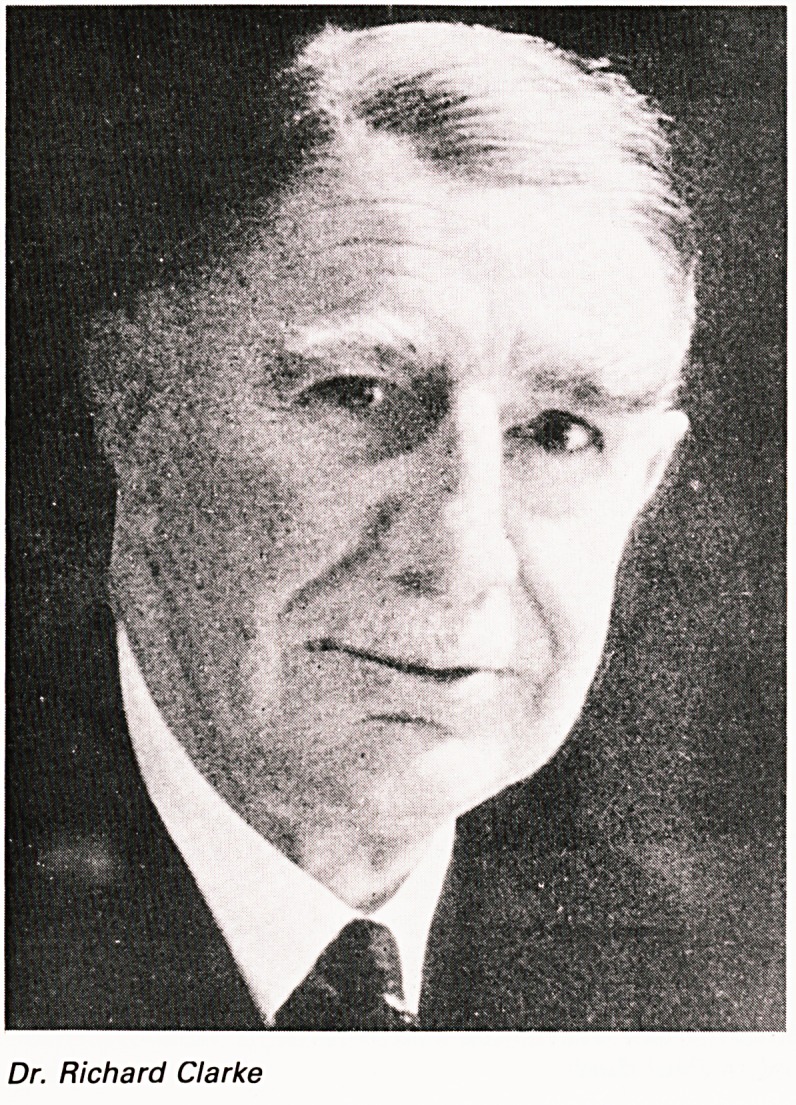


**Figure f7:**
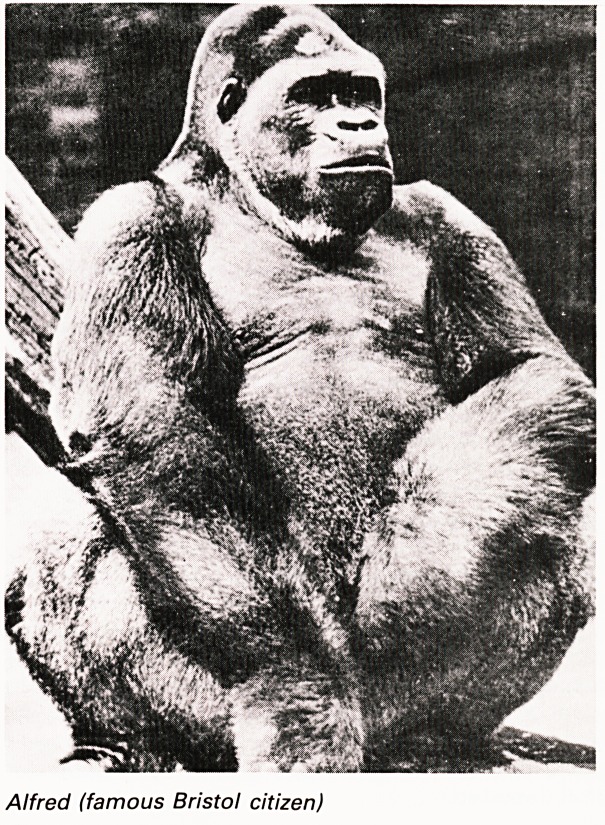


**Figure f8:**
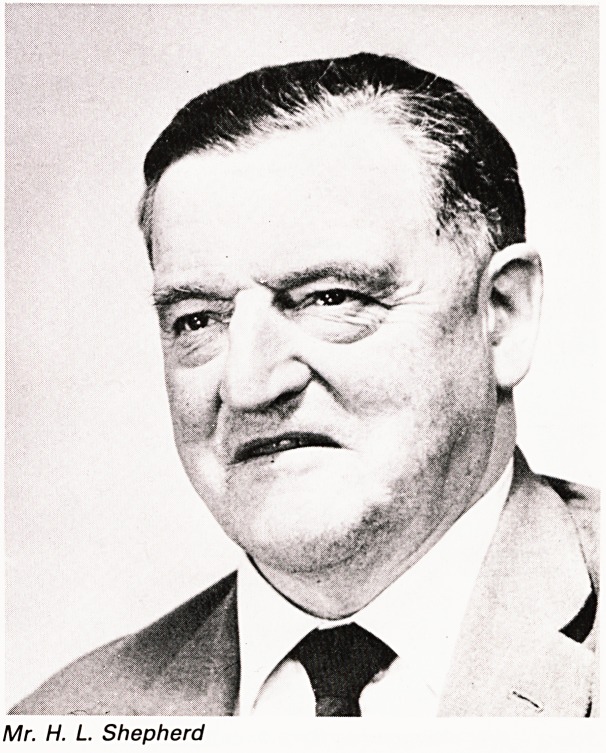


**Figure f9:**
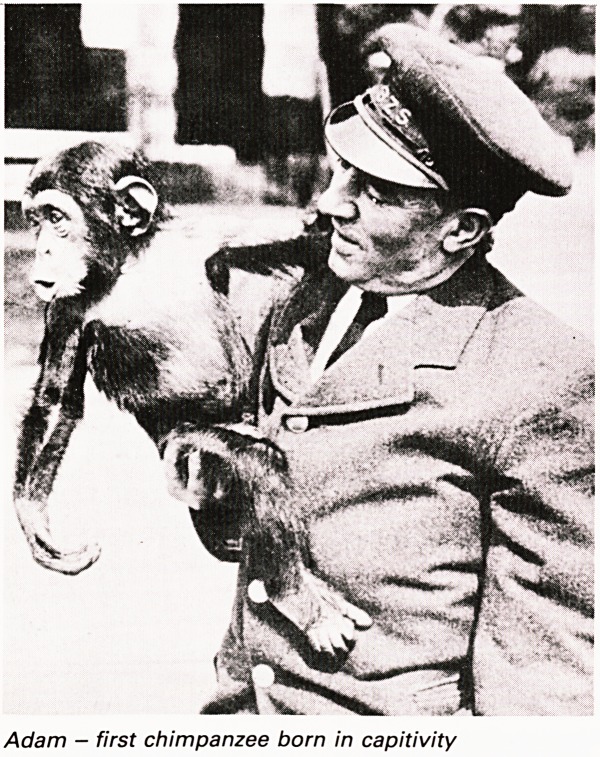


**Figure f10:**
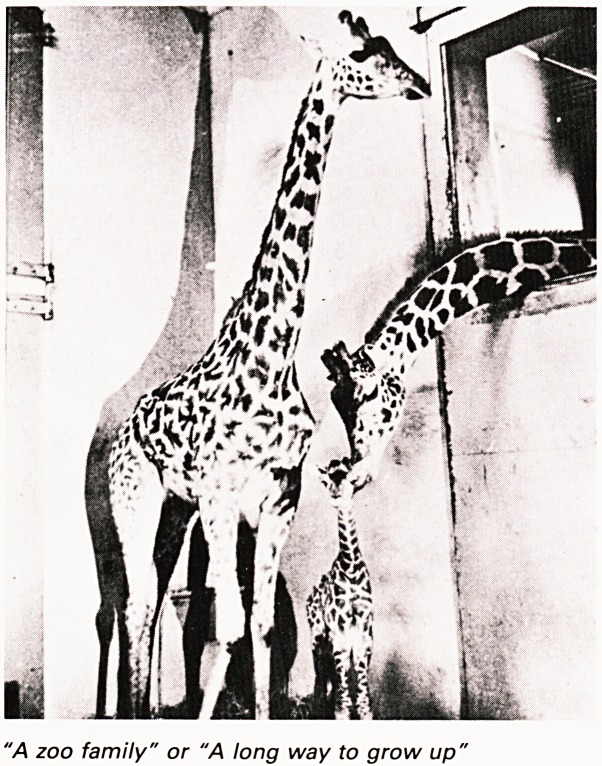


**Figure f11:**
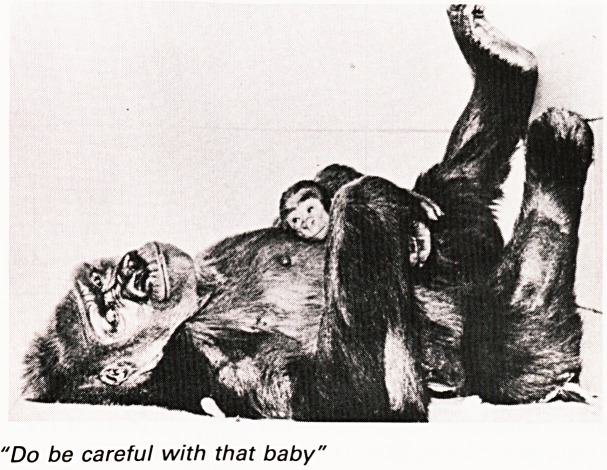


**Figure f12:**
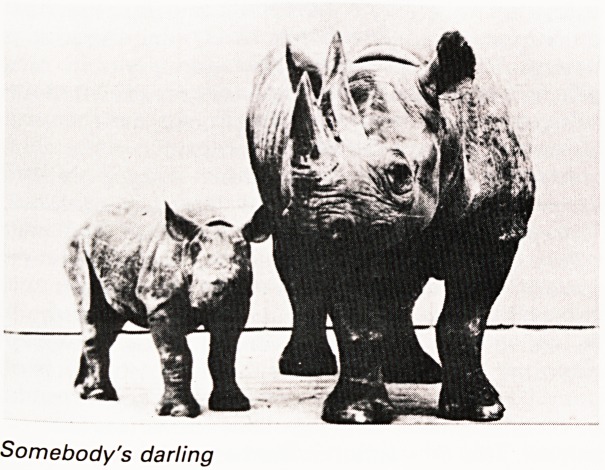


**Figure f13:**
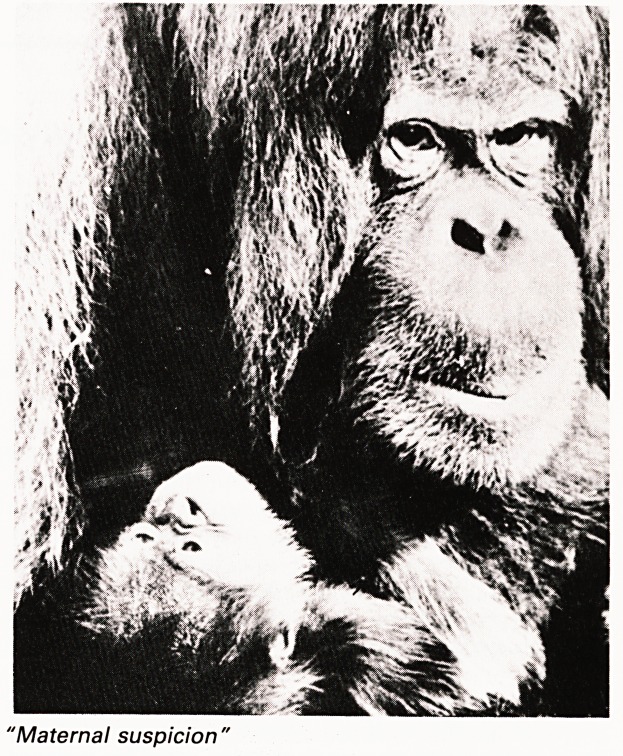


**Figure f14:**